# Vapor pressure measurements on Δ^9^-tetrahydrocannabinol, cannabidiol, and cannabinol to inform cannabis breathalyzer development

**DOI:** 10.1088/1752-7163/ae3794

**Published:** 2026-01-30

**Authors:** Cheryle N Beuning, Jennifer L Berry, Eugene Paulechka, Marcia L Huber, Kavita M Jeerage, Jason A Widegren, Tara M Lovestead

**Affiliations:** Applied Chemicals and Materials Division, Material Measurement Laboratory, National Institute of Standards and Technology, 325 Broadway, Boulder, CO 80305, United States of America

**Keywords:** cannabinoid vapor pressures, dynamic vapor microextraction, Δ^9^-tetrahydrocannabinol (THC), cannabidiol (CBD), cannabinol (CBN), aerosol-vapor partitioning, cannabis breathalyzer

## Abstract

Δ^9^-tetrahydrocannabinol (THC), the main psychoactive compound in cannabis, and other drug molecules that have large molar masses, are often described as ‘nonvolatile’ and are presumed to be carried in exhaled breath aerosols. Large variabilities in THC concentrations in breath have been measured with devices that only collect aerosols; it is possible that neglecting the vapor phase could be responsible. Partitioning of compounds between vapor and aerosol phases is directly dependent on vapor pressure (*p*_sat_), which itself is strongly dependent on temperature. We describe *p*_sat_ measurements for THC, cannabidiol (CBD), and cannabinol (CBN) using a gas-saturation apparatus. The measured values of *p*_sat_ for 364 K to 424 K are 0.0459 Pa to 7.833 Pa for THC, 0.0826 Pa to 13.44 Pa for CBD, and 0.0199 Pa to 5.678 Pa for CBN. The combined standard (*k*= 1, 68% confidence) measurement uncertainty in *p*_sat_ ranges from 2.9% to 5.3% for CBD and CBN, and from 5.2% to 9.5% for THC. To obtain the *p*_sat_ at human body and exhaled breath temperatures, we extrapolated the measurements for each cannabinoid with a thermodynamic correlation. Then a vapor-aerosol partitioning model was used to predict mole fractions of each cannabinoid in each phase of exhaled breath. All three cannabinoids were predicted to reside primarily in the vapor phase of exhaled breath. However, relatively small changes in temperature or aerosol concentration can significantly impact the predicted partitioning. This work illustrates the utility of low-uncertainty *p*_sat_ measurements for any drug, including those thought to be too low in volatility for vapor-phase sampling, and may extend the market for forensic drug tests and clinical diagnostic tests via breath analysis.

## 1. Introduction

Breath-based detection of intoxicating molecules is attractive for law enforcement to use at the roadside. The first ethanol breathalyzer was developed in the 1930s and used color change as an indicator of the reaction of ethanol with potassium permanganate [1]. Since that beginning, the precision and sensitivity of ethanol breathalyzers has improved to reliably detect breath ethanol concentrations and aid in enforcing driving laws.

**Figure 1.**
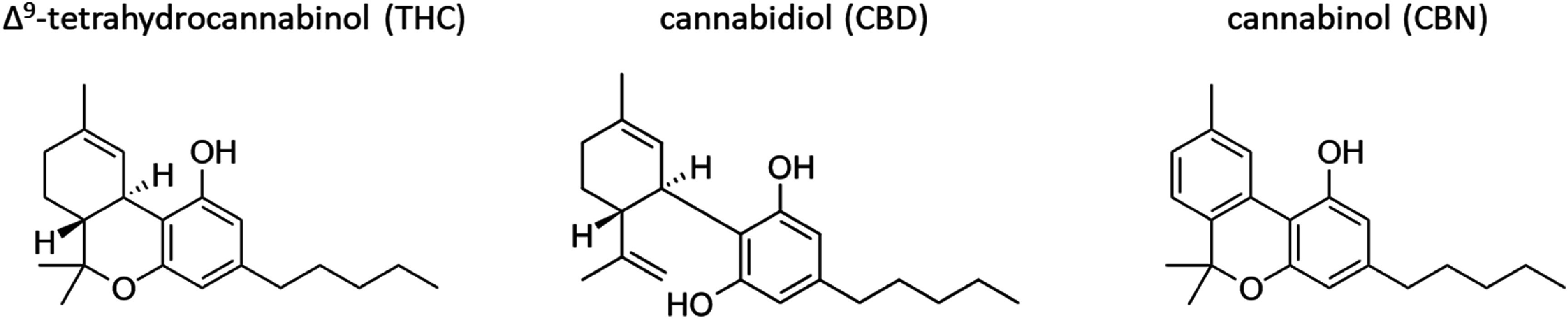
Chemical structures of the cannabinoids in this study.

Δ^9^-tetrahydrocannabinol (THC) is the main psychoactive component in cannabis and has been the target molecule for cannabis breathalyzer development for law enforcement and workplace drug detection. In contrast to ethanol, accurate determination of THC concentration in breath is currently difficult to accomplish. This is in part because a standard alcoholic drink contains about 10 g of ethanol, while a single ‘serving’ of cannabis is typically defined to contain only about 10 mg of THC [2]. Additionally, cannabis plants produce many cannabinoids, some of which are very similar to THC, which creates concerns about measurement interference. Figure 1 shows that chemical structures of THC (CASRN 1972-08-3), cannabidiol (CBD, CASRN 13956-29-1), and cannabinol (CBN, CASRN 521-35-7). THC and CBD are structural isomers with the same molecular formula (C_21_H_30_O_2_; 314.47 g⋅mol^−1^). THC and CBN (C_21_H_26_O_2_; 310.47 g⋅mol^−1^) differ only by the level of unsaturation in the first ring structure. These small changes in structure impart extremely different activities in the body; both CBD and CBN are not intoxicating. The passage of the Farm Bill [3] in 2018 effectively legalized CBD in the United States if it is derived from hemp; numerous CBD-infused products (oils, gummies, beverages, and more) are now legally available. CBN arises naturally in cannabis plants and concentrations can increase as THC degrades to CBN during drying, storing, or heating of cannabis flowers or products [4]. Both CBD and CBN have been detected in the breath of study participants that use cannabis products [5].

To date, breath sampling studies on cannabis users have largely ignored the vapor phase of exhaled breath [5]. THC, CBD, and CBN, together with other drug molecule classes like opioids [6], are often described as ‘nonvolatile’ molecules that are assumed to have no partitioning into the vapor phase at equilibrium, and thus are presumed to be carried out of the lungs only in breath aerosols. These aerosol particles are formed in the lungs by the mechanical action of breathing. Specifically, the collapse and subsequent reopening of peripheral airways causes the fluid film that lines the respiratory tract to rupture, resulting in aerosols that consist primarily of phospholipids [7–9]. Devices designed to trap drug-laden aerosols from exhaled breath have incorporated fibrous filters or impaction filters, and these devices have indeed recovered THC from breath after cannabis inhalation [5, 10–15]. Only one study utilized a device that traps aerosol particles via sedimentation during the condensation of breath vapor, thus collecting both phases of breath [16]. All these studies reveal high variability and very low concentrations for THC in breath samples (both within and between studies), which is problematic for the implementation of cannabis breathalyzers in a legal setting. Consequently, it is unlikely that cannabis breathalyzer development will flourish without a better understanding of the underlying measurement principles.

One obvious source of measurement uncertainty comes from breath sampling. Alcohol breathalyzers measure ethanol concentration in the end expiratory portion of a single breath in real time. On the other hand, because of the dramatically lower THC concentrations, prototype cannabis breathalyzers operate by preconcentrating THC from multiple full exhalations for later analysis. Simulations show that the trapping efficiency of impaction filters depends strongly on flow rate through the filters [17]. Flow rate is also known to affect the performance of devices that rely on sedimentation or impingement [18, 19].

Another source of measurement uncertainty could arise from ignoring the vapor phase of exhaled breath. In the atmosphere, compounds with varying volatility are known to interact with aerosols (e.g. dust, smoke, or secondary organic aerosols), and theory describing these interactions can be used to estimate the partitioning of THC between the aerosol phase and the vapor phase. A commonly used volatility classification scheme for this type of aerosol partitioning [20–22] includes volatile organic compounds (VOCs), intermediate-volatility organic compounds (IVOCs), semi-volatile organic compounds (SVOCs), and low-volatility organic compounds (LVOCs). At ambient conditions, compounds that fall within the VOC or IVOC range partition almost entirely into the vapor phase at equilibrium, compounds that fall within the SVOC range have significant mass fractions in both the aerosol phase and the vapor phase, and compounds in the LVOC range (or lower) partition almost entirely into the aerosol phase [21]. Calculations for the fraction of a substance in the aerosol phase versus the vapor phase are based on the effective saturation concentration of a substance (i.e. the concentration of organic aerosol required for a compound to be equally in the vapor and aerosol phases at specified conditions). Effective saturation concentration, in turn, is calculated from a compound’s vapor pressure at the temperature of interest [20–23].

Limited vapor pressure measurements have been reported [24] for THC and CBD, but no previously reported data exist for CBN. The enthalpy of vaporization of THC was recently determined by correlation gas chromatography and its vapor pressure was estimated [25]. Some vapor pressure data for cannabinoids is tabulated elsewhere [26], but the sources of that data are not specified. Simple models are available [27–32] that can be used to predict the vapor pressure of a molecule based on atomic connectivity—such models can, of course, be applied to cannabinoids. For THC and CBD vapor pressures have been predicted by molecular simulation [33]. The predicted values of vapor pressure for cannabinoids differ widely between models and, in the absence of low-uncertainty measurements, there is no way judge the accuracy of the predictions.

The primary reason for the poor data situation for cannabinoids (as described in the previous paragraph) is that it is difficult to measure the vapor pressure of such large molecules. At lower temperatures, the vapor pressures of large molecules are very small, which leads to large relative uncertainties in the measurements. At higher temperatures, thermal degradation of the sample can cause large systematic errors in the measurement. To deal with this stability limitation, we recently developed a vapor pressure measurement method based on dynamic vapor microextraction (DVME), which allows for relatively high-temperature measurements by decreasing the total measurement period [34]. DVME-based vapor pressure measurements have been published for the reference compound *n*-eicosane [34] (C_20_H_42_; 282.56 g⋅mol^−1^) and the cannabis-associated terpenoid linalool [35] (C_10_H_18_O; 154.25 g⋅mol^−1^).

Herein, we report DVME-based vapor pressure measurements from 364 K to 424 K for THC, CBD, and CBN. A vapor-pressure correlation is then used to extrapolate vapor pressures for each cannabinoid to human body temperature (310.15 K) and breath temperature (307.15 K). These extrapolated values and an aerosol partitioning model [20–23] are used to estimate the fraction of each cannabinoid in the vapor and aerosol phases of breath.

## 2. Materials and methods

### 2.1. Analysis with gas chromatography with flame ionization detection (GC-FID)

An Agilent^1^1Specific commercial equipment, instruments, or materials are identified in this paper to specify the experimental procedure adequately. Such identification is not intended to imply recommendation or endorsement by NIST, nor is it intended to suggest that the materials or equipment identified are necessarily the best available for the purpose. Contribution of the National Institute of Standards and Technology, not subject to copyright in the US. 6890 GC-FID with a DB-17 MS column (15 m length, 0.25 mm inner diameter, 0.25 *μ*m film thickness of 50% phenyl-methylpolysiloxane) was used to analyze chemical purity and eluent samples. Inlet parameters for 1.0 *μ*l splitless injections were at an inlet temperature of 300 °C, an inlet pressure of 10.0 psi (69 kPa), an N_2_ flow of 53.1 ml⋅min^−1^, and a purge flow of 50 ml⋅min^−1^ for 0.8 min. The oven temperature started at 225 °C, was ramped to 300 °C over 5 min (15 °C⋅min^−1^), and was held at 300 °C for 1 min, for a total run time of 6 min. The FID was set at 325 °C with H_2_ flow at 35 ml⋅min^−1^, air flow at 300 ml⋅min^−1^, and a N_2_ makeup flow of 25 ml⋅min^−1^. This method was developed from a published procedure [36].

### 2.2. Chemicals

**Figure 2.**
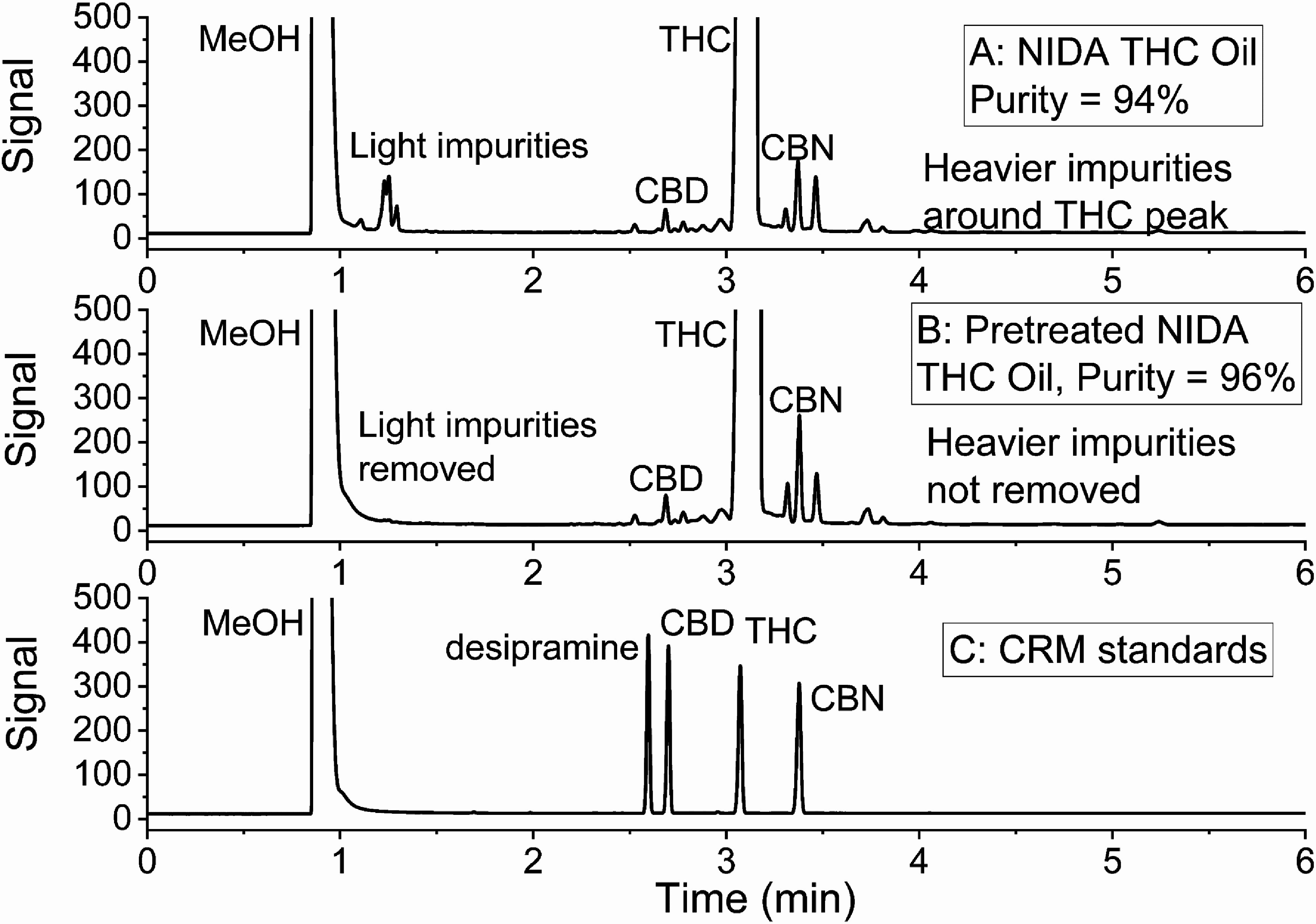
GC-FID chromatograms of NIDA THC oil after (A) vacuum concentration of ethanol solutions and (B) pretreatment by flowing 1000 scc of helium through a saturator vial at 424 K. Panel (C) shows peaks for CRMs of desipramine, CBD, THC, and CBN. The *y*-axis scale was set to show small impurity peaks.

A complete list of compounds used in this study, along with their CAS registry number, grade, supplier, and purity is available in table S1 of the supporting information. Helium (>99.999%), methanol (99.9%), *n*-eicosane (⩾99.5%), and *n*-octadecane (⩾99.0%) were obtained from commercial sources. Certified reference materials (CRMs) containing THC, CBD, CBN, and desipramine HCl [36] were obtained from Cerilliant Corporation (Round Rock, Texas, USA) as methanol solutions with nominal concentrations of 1 mg⋅ml^−1^. Gram quantities of THC, CBD, and CBN were obtained from the National Institute on Drug Abuse (NIDA, USA) through a Schedule 1 Researcher Controlled Substance Registration with the Drug Enforcement Agency. As received, NIDA CBD was a light-yellow solid with a purity of 97.7% as determined by GC-FID (based on raw peak areas). NIDA CBN was a light-orange solid with a purity of 99.1% as determined by GC-FID. NIDA THC was received in 5 ml ethanol solutions with a nominal concentration of 20 mg⋅ml^−1^. THC solutions were vacuum concentrated in silanized glass vials at 308 K until dry (∼1.5 h) and stored at 253 K. Figure 2(A) shows a GC-FID chromatogram of the resulting amber-colored THC with a purity of 94.0%. Both light impurities and heavier impurities around the THC chromatographic peak were observed. Figure 2(C) shows the chromatogram of the cannabinoid CRMs, which reveal that the heavier impurities around the THC peak from the THC oil in figure 2(A) include CBD and CBN. Prior to making vapor pressure measurements, the THC oil in the saturator vial was pretreated at 424 K for 20 min with 50 standard cubic centimeters per minute (sccm) of helium flow, for a total flow of 1000 standard cubic centimeters (scc) of helium. A representative GC-FID chromatogram in figure 2(B) shows that pretreatment removed the light impurities. After pretreatment, the color of the THC oil in the saturator vial changed from amber to a much lighter yellow, indicating that the volatile impurities accounted for most of the color in the vacuum-concentrated THC. Importantly, when allowed to cool to room temperature, the pretreated THC oil solidified, which is an unequivocal indication of higher purity, which was measured by GC-FID to be 96.0%.

### 2.3. DVME apparatus

**Figure 3.**
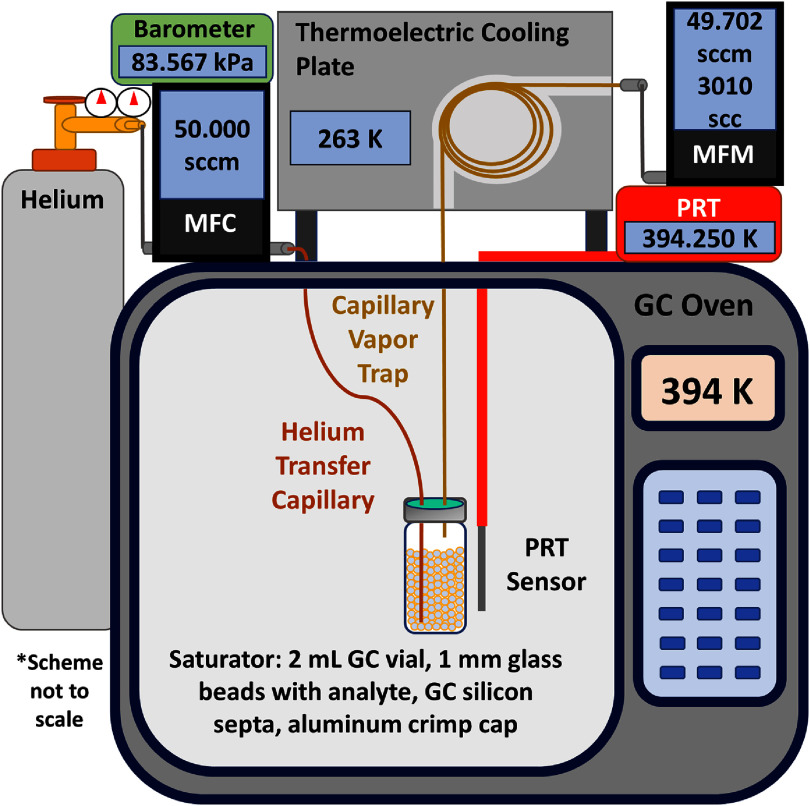
The dynamic vapor microextraction (DVME) apparatus for rapid cannabinoid vapor pressure measurements. The apparatus consists of a helium supply cylinder with regulator; a helium mass flow controller (MFC); a helium transfer capillary between the MFC and the saturator; a saturator made from a 2 ml autosampler vial that is crimp capped with a silicone septum and filled with 1 mm glass beads coated in analyte; a capillary vapor trap secured to a thermoelectric cooling plate; a helium MFM; and a platinum resistance thermometer (PRT) to measure temperature. A barometer was used to determine the overpressure in the saturator in separate experiments.

The overall design of the DVME apparatus (figure 3) is substantially similar to earlier versions used to measure the vapor pressures of *n*-eicosane [34] and linalool [35]. However, for the thermally labile cannabinoids, we were concerned about chemical degradation during the measurements, which must run for the time necessary to collect a sufficient mass of cannabinoid in the capillary vapor trap. We decreased measurement time by both increasing the helium carrier gas flow rate and the temperature for the vapor pressure measurements. We increased the helium gas flow rate from 10 sccm to 50 sccm. We increased the measurement temperature range from 344 K–374 K to 364 K–424 K. The higher temperatures and higher flow rates both significantly increase overpressure in the saturation vial during measurements. This overpressure is caused by viscous flow of the carrier gas through the capillary vapor trap. In this work, we incorporated capillaries with a larger internal diameter (ID) to reduce the magnitude of the effect. Specifically, the helium transfer capillary (figure 3) is a 0.5 m length of deactivated fused silica with an ID of 530 *μ*m and the capillary vapor trap is a 1.0 m length of DB-35MS (35%-phenyl-methylpolysiloxane) GC column with a film thickness of 0.5 *μ*m and an ID of 530 *μ*m. We also specifically account for overpressure in the vapor pressure calculation (equations (1)–(4) below) to eliminate a systematic measurement error. We also removed the previously used helium transfer vial, which allowed us to decrease the overall length of capillary tubing and to decrease the void volume. See section C, figure S2 of the supplementary information for details on the overpressure measurements. A mass flow controller (MFC) and mass flow meter (MFM) with higher full-scale flows for low uncertainty operation in the increased flow rate regime were used. Aluminum crimp caps and a silicone GC inlet septum were used as the saturator cap to mitigate leaking at the previously used screw caps with standard PTFE-silicone septa. As received, the GC inlet septa are too thick to fit with the aluminum cap, so we halved the thickness by slicing it at the perimeter indentation with a scalpel. The design modifications were validated with *n*-eicosane; these results are presented below in section 3.1.

### 2.4. Experimental method for vapor pressure

New saturators were prepared daily with new glass beads freshly coated with cannabinoid. For saturators containing CBD or CBN, ∼60 mg of the solid-phase sample from NIDA was placed with the glass beads in the saturator. The CBD or CBN was then melted at 363 K for 10 min and the mixture stirred to evenly coat the glass beads with the liquid-phase cannabinoid. The saturator was then capped and installed in the DVME apparatus (figure 3). The capillary vapor trap was secured with most of it mounted to the thermoelectric cooling plate (set at 263 K) with aluminum tape and insulating polystyrene foam. One end of the capillary vapor trap connects to the saturator vial (housed in the oven) and the other end connects to the MFM. For saturators containing THC, ∼60 mg of the vacuum-concentrated THC oil was deposited on the inner walls of the vial with a glass pipette. Then the glass beads were added to the vial, and the glass pipette was used to stir the beads and oil to fully coat the beads. The saturator was capped and installed in the DVME apparatus, along with a capillary vapor trap.

Each day, a series of vapor pressure measurements were made from 364 K to 424 K in 10 K steps, from the lowest to the highest temperature. For each cannabinoid, this temperature series was repeated six times (three days per week for two consecutive weeks). For the first three days of measurements on each cannabinoid, the 364 K measurement was repeated after the full temperature ramp to check for sample degradation. The thermal equilibration period at the beginning of each measurement (during which the flow rate of helium through the capillary was only 0.2 sccm) was 3 min for THC measurements, but this was increased to 6 min for CBD and CBN measurements to decrease the uncertainty in measurement temperature (see below). For all measurements on the cannabinoids, the helium flow rate was 50 sccm; however, to compensate for less cannabinoid collection in the vapor trap at lower temperatures, the total flow of helium was increased. For measurements at 424 K, the total helium flow was about 1200 scc (∼24 min). For measurements at 404 K and 414 K, the total helium flow was about 2000 scc (∼40 min). For measurements from 364 K to 394 K, the total helium flow was about 3000 scc (∼60 min). For measurements on CBN at 364 K, the lowest vapor pressures were observed, and the total helium flow was doubled to about 6000 scc (∼120 min).

After stopping the helium flow, the capillary vapor trap was removed from the DVME apparatus and immediately rinsed with 0.8 ml of methanol to remove trapped cannabinoid. The eluent was collected in a silanized amber autosampler vial that contained approximately 0.16 g of the ∼1 mg⋅ml^−1^ solution of desipramine HCl in methanol [36]. The capillary vapor trap was eluted again with an additional 1.0 ml of methanol into a second silanized amber autosampler vial, and both eluent fractions were analyzed by GC-FID. Cannabinoid analyte was never observed in the second eluent fraction. The cannabinoid mass in the first eluent fraction was determined from the linear relationship of mass ratio versus area ratio for the specific cannabinoid and the internal standard. Details of the sensitivity analysis with the cannabinoid CRMs are given in section B of the supporting information.

### 2.5. Calculations and measurement uncertainty

Equations (1)–(4) were used to calculate the vapor pressure, *p*_sat_, for each cannabinoid [34, 35, 37],

\begin{align*}{p_{{\mathrm{sat}}}} &amp;= {p_{{\mathrm{saturator}}}} \cdot {y_2}/{x_2}\end{align*}

\begin{align*}{p_{{\mathrm{saturator}}}} &amp;= {p_{{\mathrm{ambient}}}} + {p_{{\mathrm{overpressure}}}}\end{align*}

\begin{align*}{y_2} &amp;= \frac{{{m_2}/{M_2}}}{{\left( {{m_1}/{M_1} + {\text{ }}{m_2}/{M_2}} \right)}}\end{align*}

\begin{align*}{x_2} &amp;= 1 - {x_1} - {x_{{\mathrm{impurities}}}}\end{align*}

where the subscript ‘1’ indicates values for helium and the subscript ‘2’ indicates values for THC, CBD, or CBN. Briefly, *p*_sat_ was calculated with equation (1) from the pressure measured in the saturator (*p*_saturator_), the mole fraction of cannabinoid calculated in the vapor phase (*y*_2_), and the purity (as a mole fraction) of cannabinoid in the condensed phase (*x*_2_), which is measured with GC-FID. The value of *p*_saturator_ was calculated with equation (2) as the sum of the ambient pressure (*p*_ambient_) and the overpressure (*p*_overpressure_). The overpressure was measured for each temperature at a flow rate of 50 sccm [34, 35], see section C of the supporting information for more details. The value of *y*_2_ was calculated with equation (3) from the total mass of helium (*m*_1_, measured by the MFM), the mass of cannabinoid recovered from the capillary vapor trap (*m*_2_, measured by GC-FID), the molar mass of helium (*M*_1_), and the molar mass of the cannabinoid (*M*_2_). Last, the value of *x*_2_ for each cannabinoid was calculated from equation (4), where *x*_1_ is the mole fraction solubility of helium in liquid cannabinoid and *x*_impurities_ is the mole fraction of impurities measured by GC-FID. Values of *x*_1_ have not been reported; nevertheless, given helium’s low solubility in other organic liquids (such as *n*-eicosane [34]), it is safe to assume that *x*_1_ is negligible. The uncertainty analysis and error propagation calculations for experimental variables used to calculate *p*_sat_ with equations (1)–(4) is detailed in section D of the supporting information.

The experimental data were fitted with the Clarke–Glew equation [38] truncated to the heat-capacity term:

\begin{align*}R{\mathrm{ln}}\left( {\frac{{{p_{{\mathrm{sat}}}}}}{{{p_0}}}} \right) &amp;= - \frac{{{\Delta _{{\mathrm{vap}}}}G^\circ \left( \theta \right)}}{\theta } + {\Delta _{{\mathrm{vap}}}}H^\circ \left( \theta \right)\left( {\frac{1}{\theta } - \frac{1}{T}} \right)\nonumber\\ &amp;\quad + {\Delta _{{\mathrm{vap}}}}{C_p}^\circ \left( \theta \right)\left( {\frac{\theta }{T} - 1 + {\mathrm{ln}}\left( {\frac{T}{\theta }} \right)} \right){\text{ }}\end{align*}

where *p*_0_ = 0.1 MPa, ${\Delta _{{\mathrm{vap}}}}G^\circ \left( \theta \right)$ and ${\Delta _{{\mathrm{vap}}}}H^\circ \left( \theta \right)$ are the standard Gibbs energy and enthalpy of vaporization at the reference temperature *θ* = 307.15 K. The heat-capacity change was assumed to be independent of temperature and equal to that at *T* = 298.15 K. The latter was estimated using the correlation by Chickos *et al* [39] in the form:

\begin{align*}{\Delta _{{\mathrm{vap}}}}{C_p}^\circ /\left( {{\mathrm{J}} \cdot {{\mathrm{K}}^{{{- 1}}}} \cdot {\mathrm{mo}}{{\mathrm{l}}^{{{- 1}}}}} \right) &amp;= - 14.30 - 0.35{C_p}^\circ \left( {\mathrm{g}} \right)\nonumber\\ &amp;\quad/\left( {{\mathrm{J}} \cdot {{\mathrm{K}}^{{{- 1}}}} \cdot {\mathrm{mo}}{{\mathrm{l}}^{{{- 1}}}}} \right)\end{align*}

*C_p_*°(g) for all three compounds was found using the statistical thermodynamic model published recently [40]. The expanded uncertainty of these values was estimated to be 0.02 *C_p_*°(g). The expanded uncertainty of ${\Delta _{{\mathrm{vap}}}}{C_p}^\circ $ was assumed [39] to be 0.2${\Delta _{{\mathrm{vap}}}}{C_p}^\circ $.

## 3. Results and discussion

### 3.1. Measurement validation with n-eicosane

Compared to vapor pressure measurements made previously with the DVME apparatus, *n*-eicosane [34] and linalool [35], cannabinoids have lower vapor pressures. Consequently, to collect enough cannabinoid in the capillary vapor trap for a low-uncertainty analysis by GC-FID, we had to use higher measurement temperatures (from 364 K to 424 K) and larger total flow volumes of helium (from 1200 scc to 6000 scc). However, given that THC and CBD can begin to decompose during longer measurement times [24], we kept the measurement period as short as possible by using a higher helium flow rate (50 sccm). As described in section 2.3, multiple minor hardware changes to the components of the DVME apparatus were made to accommodate the higher temperatures and flow rate.

The new DVME configuration was validated with vapor pressure measurements on *n*-eicosane. In addition to having measured *n*-eicosane with the previous DVME configuration, *n*-eicosane has the lowest-uncertainty vapor pressure measurements [34, 41] for a molecule its size, and it has a reference correlation [42] that facilitates measurement comparisons. Additionally, *n*-eicosane is thermally stable and available commercially in high purity. Thus, *n*-eicosane is the best choice for measurement validation [34] for molecules similarly sized to cannabinoids. Vapor pressure measurements on *n*-eicosane were made in triplicate at 373.91 K with a flow rate of 50 sccm. The *p*_sat_ values measured were (5.427 ± 0.137) Pa, (5.766 ± 0.146) Pa, and (5.399 ± 0.137) Pa, with combined standard uncertainties [34] of *u*_c_(*p*_sat_) = 0.0253·*p*_sat_. For comparison, the reference correlation of Lemmon and Goodwin [42] predicts a value of (5.636 ± 0.192) Pa at 373.91 K, where the standard uncertainty in the correlation was estimated as before [34]. The standard uncertainty intervals of the measurements and the reference correlation overlap in every case, which is evidence that the newly configured DVME apparatus can operate at higher temperatures and a higher flow rate without significant impacts to accuracy. With the new configuration, cannabinoid analyte vapor breakthrough was investigated by replacing the MFM with a GC vial filled with solvent to check for cannabinoid under equivalent experimental method conditions. No cannabinoid breakthrough was observed. section E of the supporting information provides more details.

Our experimental design includes running a complete series of vapor pressure measurements from 364 K to 424 K in 10 K steps, and then repeating the measurement at the lowest temperature, 364 K, in one day with the same saturator. This last measurement was used to check for repeatability with the first 364 K measurement of the day and is the most reliable indicator that sample degradation is not affecting the *p*_sat_ measurements. The remaining cannabinoid in the saturator was dissolved in methanol and its purity analyzed by GC-FID. For each measurement series, comparison of as-received purity (or, in the case of THC, purity after pretreatment) with purity after the 364 K repeat measurement showed no significant cannabinoid thermal instability, table 1.

**Table 1.** Cannabinoid percent purities were determined by GC-FID and calculated as 100% (area of cannabinoid peak)/(area of cannabinoid peak + area of impurity peaks). The initial purity (after pretreatment in the case of THC) is given, along with the purity of the cannabinoid that remained in the saturator at the end of each series of *p*_sat_ measurements. The standard uncertainty in the peak area fraction is estimated to be 0.4% for THC, 0.3% for CBD, and 0.1% for CBN.

	Purity of THC	Purity of CBD	Purity of CBN
Initial	96.0%	97.7%	99.1%
After Series 1	95.4%	96.9%	99.1%
After Series 2	95.9%	97.0%	99.1%
After Series 3	96.1%	97.1%	99.1%
After Series 4	96.4%	97.0%	99.1%
After Series 5	95.8%	96.8%	99.1%
After Series 6	95.7%	96.8%	99.1%

### 3.2. Vapor pressures and uncertainty for THC, CBD, and CBN

Measurements of *p*_sat_ were conducted for THC, CBD, and CBN at seven nominal temperatures from 364 K to 424 K. Table 2 presents the combined standard (*k*= 1, 68% confidence) measurement uncertainty in vapor pressure, *u*_c_(*p*_sat_), for each cannabinoid at each nominal temperature. Values for *p*_sat_ and *u*_c_(*p*_sat_), along with all the variables used to calculate *p*_sat_ and the standard uncertainty in each variable, are presented in section F of the supporting information. An uncertainty budget for each cannabinoid is available in section G of the supporting information. At the lower measurement temperatures, the largest source of measurement uncertainty is from the determination of *m*_2_, which is a result of the relatively small amount of cannabinoid deposited in the capillary vapor trap for these measurements. At the higher measurement temperatures, the largest source of measurement uncertainty is from uncertainty in *T*, which is primarily caused by incomplete temperature equilibration at the beginning of helium flow. Both of these sources of measurement uncertainty could be decreased by increasing the measurement period, but we chose not to do this because sample degradation has been previously observed with longer measurement periods [24]. For CBD and CBN, the value of *u*_c_(*p*_sat_) ranges from about 3% to approximately 5% of the measured value. For THC, the value of *u*_c_(*p*_sat_) ranges from about 5% to almost 10% of the measured value. The larger values of *u*_c_(*p*_sat_) for the THC measurements are primarily caused by the use of a shorter thermal equilibration period (3 min for THC vs 6 min for CBD and CBN), which increases the standard uncertainty in the measurement temperature, *u*(*T*), as shown in table S6 of the supporting information.

**Table 2.** The combined standard (*k* = 1, 68% confidence) uncertainty in *p*_sat_, *u*_c_(*p*_sat_), for each cannabinoid at each nominal measurement temperature.

Temperature	*u*_c_(*p*_sat_)THC	*u*_c_(*p*_sat_)CBD	*u*_c_(*p*_sat_)CBN
364 K	0.052∙*p*_sat_	0.034∙*p*_sat_	0.053∙*p*_sat_
374 K	0.053∙*p*_sat_	0.029∙*p*_sat_	0.041∙*p*_sat_
384 K	0.058∙*p*_sat_	0.030∙*p*_sat_	0.030∙*p*_sat_
394 K	0.060∙*p*_sat_	0.030∙*p*_sat_	0.031∙*p*_sat_
404 K	0.067∙*p*_sat_	0.035∙*p*_sat_	0.037∙*p*_sat_
414 K	0.069∙*p*_sat_	0.040∙*p*_sat_	0.042∙*p*_sat_
424 K	0.095∙*p*_sat_	0.050∙*p*_sat_	0.051∙*p*_sat_

### 3.3. Vapor pressure correlations for THC, CBD, and CBN

The experimental data were fitted by equation (5) using the unweighted least-squares method to determine two parameters, ${\Delta _{{\mathrm{vap}}}}G^\circ \left( \theta \right)$ and ${\Delta _{{\mathrm{vap}}}}H^\circ \left( \theta \right)$, for each compound. Initially, all experimental results were used in the fitting. It was found that the deviation of the values at *p*_sat_ < 0.1 Pa significantly exceed the uncertainties estimated above. Therefore, only the data at *p*_sat_ > 0.1 Pa were used in the final fit. The resulting parameters are reported in table 3.

**Table 3.** The parameters of equation (5) for each cannabinoid [40].

	THC	CBD	CBN
*θ/*K	307.15	307.15	307.15
*p*_0_/kPa	100	100	100
${\Delta _{{\mathrm{vap}}}}G^\circ \left( \theta \right)$ /(kJ⋅mol^−1^)	54.75	52.84	56.73
${\Delta _{{\mathrm{vap}}}}H^\circ \left( \theta \right)$ /(kJ⋅mol^−1^)	119.00	117.16	123.12
${C_p}^\circ \left( {\mathrm{g}} \right)$ /(J⋅K^−1^⋅mol^−1^)^a^	407.3	422.3	387.8
${\Delta _{{\mathrm{vap}}}}{C_p}^\circ \left( \theta \right)$ /(J⋅K^−1^⋅mol^−1^)	−157	−162	−150

^a^ at *T* = 298.15 K.

**Figure 4.**
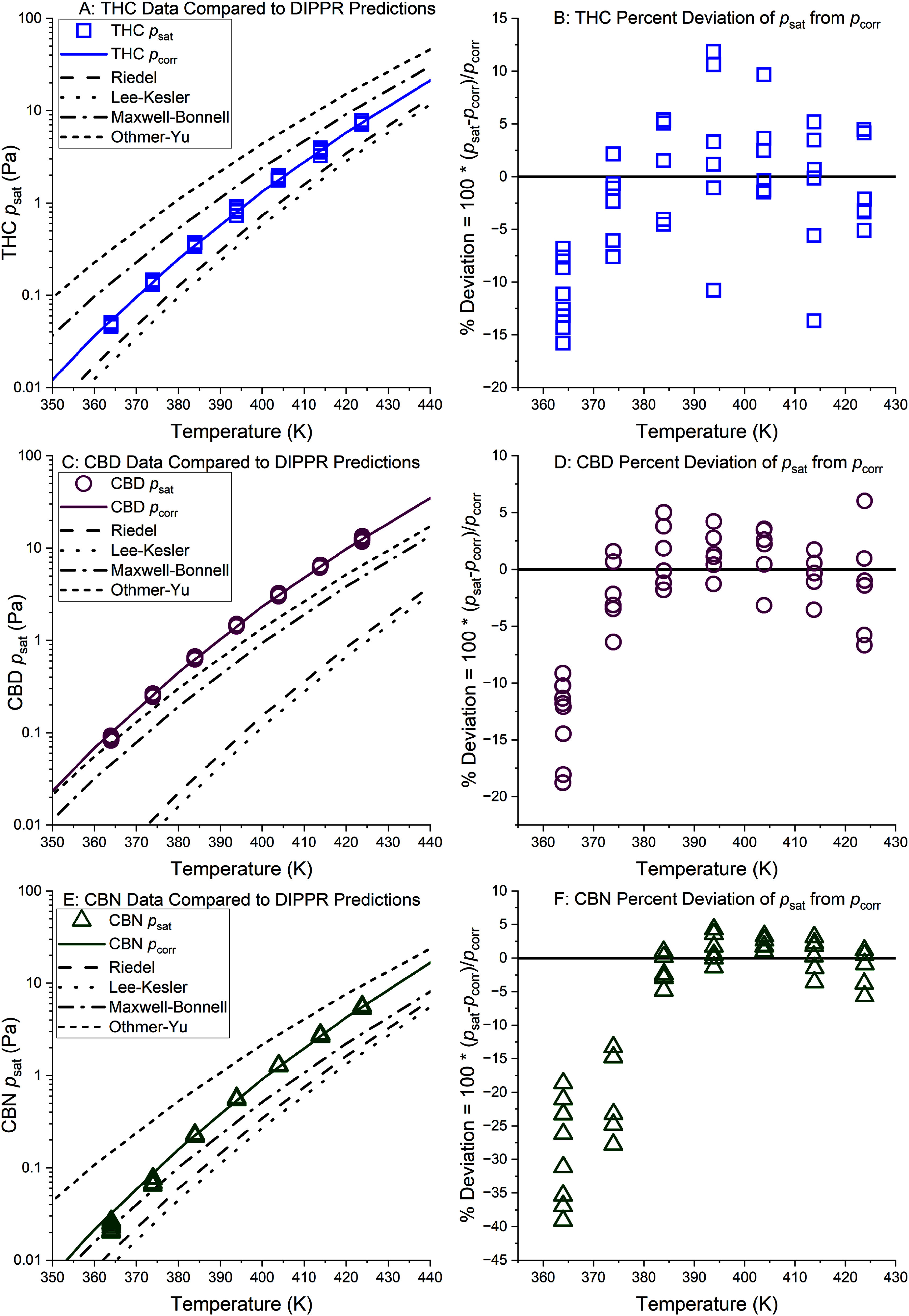
Panels (A), (C), and (E) give experimental vapor pressure data (*p*_sat_, colored symbols) used to develop a vapor pressure correlation (*p*_corr_, solid, colored line) for THC, CBD, and CBN, respectively. Four predictive models that are based on the molecular structure (dashed lines, implemented with the DIPPR database [43]) are included for comparison. Panels (B), (D) and (F) are deviation plots of *p*_sat_ from *p*_corr_ for THC, CBD, and CBN, respectively.

Panels A, C, and E in figure 4 present the experimental vapor pressure data (*p*_sat_, colored symbols) used to develop vapor pressure correlations (*p*_corr_, solid, colored lines) based on equation (5) (table 3). The predicted vapor pressure curves from models implemented in the Design Institute for Physical Properties (DIPPR) Database [43] (dashed lines, Reidel [30, 31], Lee–Kesler [27], Maxwell–Bonnell [28], and Othmer–Yu [29]) are also presented. Note that the DIPPR Database did not contain any experimental data for THC, CBD, or CBN but, even in such circumstances, it can still be used to implement predictive models based on the molecular structure (via SMILES [44], a line notation for encoding molecular structure; SMILES notation for THC, CBD and CBN are available in table S1 of the supporting information). Over the temperature range in figure 4, these predictive models differ by an order of magnitude or more and, in the case of CBD, the measured values fall outside of the range of the predictions. Clearly, the results of such simple models should be used with caution for molecules as complex as cannabinoids. There is currently no way to tell which model performs best for a given compound without low-uncertainty experimental measurements for comparison.

Panels (B), (D) and (F) in figure 4 show the percent deviation of each *p*_sat_ measurement from the *p*_corr_ curve for THC, CBD, and CBN, respectively (numerical values for *p*_corr_ and percent deviation are available in section F of the supporting information). These plots show that *p*_sat_ is systematically lower than *p*_corr_ at 364 K for all three compounds and at 374 K for CBN. As discussed above, *p*_sat_ < 0.1 Pa for all these systematically low measurements. On a related note, Panels B, D, and F in figure 4 also allow for a visual examination of scatter in repeat measurements. Except for the measurements on CBN at 364 K, the level of scatter is consistent with our estimates of *u*_c_(*p*_sat_). The extra scatter in the 364 K CBN data is likely related to the fact that these are the lowest values of *p*^sat^ measured in this work. In support of this explanation, the CBN measurements at 364 K were even more scattered before we doubled the total helium flow volume to 6000 scc to double the amount of CBN in the vapor trap, which resulted in more reliable GC-FID peak integrations. Also consistent with this explanation, CBN measurements at higher temperatures had a level of scatter like THC and CBD measurements at a similar value of *p*^sat^. In short, our methodology for estimating measurement uncertainty is likely too optimistic for measurements <0.1 Pa.

### 3.4. Comparison of p_sat_ measurements with earlier measurements and molecular dynamics simulations

**Figure 5.**
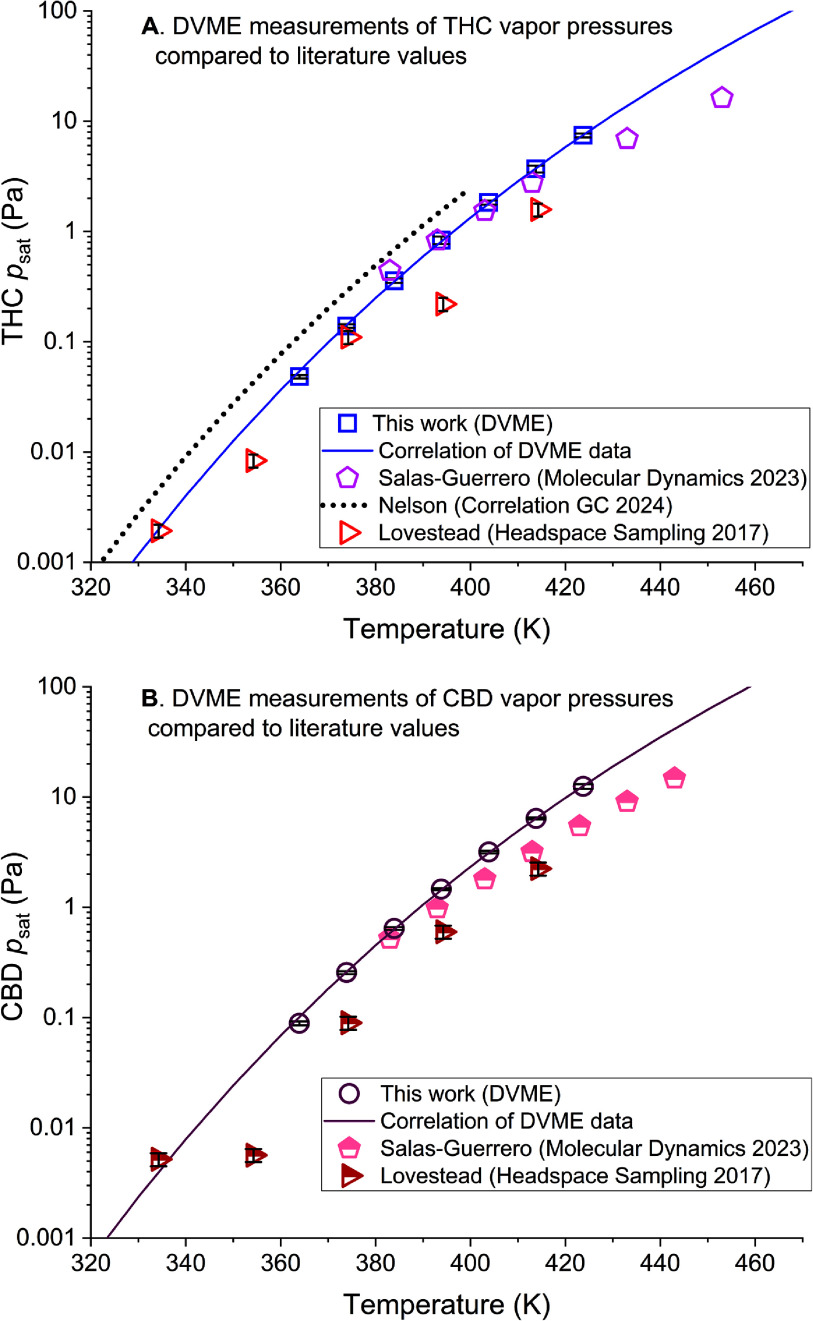
DVME measurements of vapor pressure for THC (panel A) and CBD (panel B) compared to literature values from correlation gas chromatography [25], molecular dynamics [33], and our 2017 measurements by headspace sampling [24].

In 2017, we reported *p*_sat_ of THC and CBD based on experimental measurements [24]. Notably, the headspace sampling apparatus used for those *p*_sat_ measurements inspired the development of DVME apparatus used herein. In that work, measurements on THC and CBD were made from 333 K to 413 K (in 20 K steps). Figure 5 shows a comparison of those previous measurements with the current work. The smoothness of the *p*_sat_ curve and the diminished scatter indicate the relatively high quality of the new measurements. However, the overall good agreement between the two sets of measurements is noteworthy, especially at lower temperatures. Poorer agreement at higher temperatures is likely the result of difficulty sourcing sufficient quantities of THC and CBD for the earlier study (higher loading is desirable at higher temperatures because more material evaporates) [24].

Recently, *p*_sat_ of THC was estimated between 298 K and 400 K by correlation gas chromatography [25]. Figure 5 shows that the vapor pressure curve from correlation gas chromatography matches the shape of the curve from DVME remarkably well but is, on average, about a factor of two higher than the DVME values. This offset is presumably due to the use of linear alkanes as standards.

Salas-Guerrero *et al* [33] recently published vapor pressures for THC and CBD from 383 K to 453 K that were derived from molecular dynamics simulations. The molecular dynamics predictions are all within about a factor of two of the DVME *p*_sat_ curve (figure 5), although the predicted vapor pressure curves are flatter than the measured curves, so extrapolated values would have poorer agreement. Presumably, part of the reason that this type of modeling was more successful than the simpler predictive models implemented in DIPPR is the fact that molecular dynamics simulations consider the 3D molecular structure. Vapor pressure prediction based on molecular simulation looks promising for large molecules and deserves further study.

### 3.5. Enthalpy of vaporization

The enthalpies of vaporization calculated using equation (5) are compared with literature values in table 4. Unlike *p*_sat_, the vaporization enthalpy of THC determined by correlation gas chromatography at 298.15 K is in good agreement with the value obtained in this work. The results obtained by molecular dynamics at 383 K for THC and CBD agree reasonably well with the current results at *T* = 398.8 K or 403.9 K. The temperature dependence obtained in the simulations seems to be adequate for THC but is too strong for CBD.

**Table 4.** Enthalpies of vaporization of cannabinoids^a^.

	THC	CBD	CBN
DVME apparatus, this work			

<*T*>/K	398.8	398.9	403.9
${\Delta _{{\mathrm{vap}}}}H^\circ$ (<*T*>)/(kJ⋅mol^−1^)	104.6 ± 1.4	102.3 ± 0.8	108.6 ± 0.9
${\Delta _{{\mathrm{vap}}}}H^\circ \left( {298.15{\text{ K}}} \right)$ /(kJ⋅mol^−1^)	120.4 ± 3.5	118.6 ± 3.4	124.5 ± 3.3

Correlation gas chromatography [25,45]			

${\Delta _{{\mathrm{vap}}}}H^\circ \left( {298.15{\text{ K}}} \right)$ /(kJ⋅mol^−1^)	120.4 ± 3.6		

Molecular dynamics [33]			

${\Delta _{{\mathrm{vap}}}}H^\circ \left( {383{\text{ K}}} \right)$ /(kJ⋅mol^−1^)	106.5	103.2	
${\Delta _{{\mathrm{vap}}}}H^\circ \left( {433{\text{ K}}} \right)$ /(kJ⋅mol^−1^)	97.12	80.95	

^a^ Expanded uncertainties (*k* = 2, 95% confidence) are reported.

## 4. Implications for breath analysis of cannabis users

The new vapor pressure data and the associated vapor pressure correlations allow us to perform calculations that are useful for cannabis breathalyzer development. The DVME-based *p*_sat_ measurements for THC, CBD, and CBN can be extrapolated to human body temperature (310.15 K) and exhaled breath temperature (307.15 K) with the correlations given in table 3. These correlated values of vapor pressure (*p*_corr_) are presented in table 5.

**Table 5.** Correlated values of vapor pressure (*p*_corr_) at breath and body temperatures for each cannabinoid, as determined from the vapor pressure correlations with equation (5) (table 3).

	THC	CBD	CBN
*p*_corr_ (Pa) at 307.15 K (breath)	4.9 ⋅ 10^–5^	1.0 ⋅ 10^–4^	2.3 ⋅ 10^–5^

*p*_corr_ (Pa) at 310.15 K (body)	7.7 ⋅ 10^–5^	1.6 ⋅ 10^–4^	3.6 ⋅ 10^–5^

**Figure 6.**
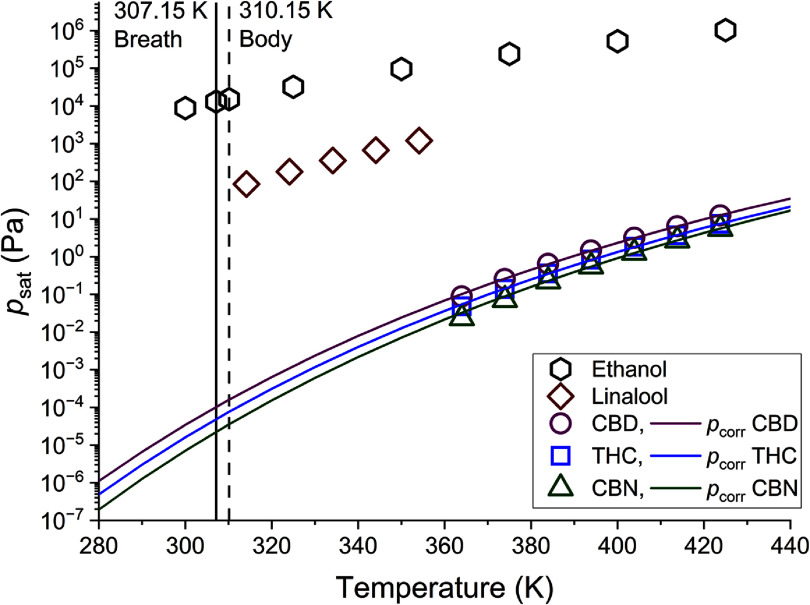
Experimental *p*_sat_ data for ethanol (hexagons, taken from TDE [50]), linalool (diamonds) [35], and data within for CBD (circles), THC (squares), and CBN (triangles). The vapor pressure correlation (from table 3), *p*_corr_, for each cannabinoid is shown as a solid line with the same color as the experimental data points. The dashed vertical line in the plot indicates human body temperature (310.15 K) and the solid vertical line represents exhaled breath temperature (307.15 K).

It is important to note that breath and body temperatures are below the triple-point temperatures for CBD (341.4 K, 341.35 K) [46, 47] and CBN (352.15 K) [48] but above the triple-point temperature of THC (280.75 K) [49], so the values of *p*_corr_ at these temperatures correspond to the supercooled liquid for CBD and CBN. However, it is unlikely that a solid phase for any cannabinoid will form in the fluid lining of the lung, so these extrapolated values are the most relevant for understanding exhaled breath. Additionally, such supercooled liquid-phase vapor pressures are used in models of organic aerosol partitioning [20, 23]. The extrapolation curves for *p*_corr_ are shown in figure 6, along with *p*_sat_ curves for the cannabis-associated terpenoid linalool [35] and for ethanol (taken from TDE [50]). An immediate conclusion from figure 6 is that, at breath temperature, *p*_corr_ for the cannabinoids is 8–9 orders of magnitude lower than the *p*_sat_ of ethanol. The approximately billion-fold lower *p*_sat_ of cannabinoids compared to ethanol is an important reason why significant investment in cannabis breathalyzer development has yet to yield a reliable roadside device for law enforcement.

The field of atmospheric chemistry uses partitioning models to predict whether an organic compound is expected to be primarily in the vapor phase, in an organic aerosol phase, or in a combination of both. In these models, the effective saturation concentration (C*) represents the concentration of organic aerosol at which a compound is equally distributed between the vapor and aerosol phases. C* can be used to group compounds of similar volatility and, if the concentration of organic aerosol is known, can be used to predict the partitioning of an organic compound between the vapor and aerosol phases. Equation (5) [20, 23],

\begin{align*}C* = \frac{{{M_2} \cdot \left( {{{10}^6}{ }\mu {\mathrm{g}} \cdot {{\mathrm{g}}^{ - 1}}} \right) \cdot \zeta \cdot {p_{{\mathrm{corr}}}}}}{{R \cdot T}},\end{align*}

was used to calculate C* (*μ*g·m^–3^) for the cannabinoids using the molar mass of the cannabinoid (*M*_2_, g·mol^–1^), a conversion from g to *μ*g, the molality-based activity coefficient of the compounds in the organic aerosol (ζ), correlated vapor pressure from this work (*p*_corr_, Pa), the universal gas constant (*R,* 8.314 m^3^·Pa·K^–1^·mol^–1^), and the temperature (*T*, K). The activity coefficients for the cannabinoids are not known but are assumed to be equal to 1, as is commonly done in these types of partitioning calculations. The resulting values of C* at breath temperature (307.15 K, table 4) are 6.0 *μ*g·m^–3^ for THC, 13 *μ*g·m^–3^ for CBD, and 2.7 *μ*g·m^–3^ for CBN. This means that, for example, for THC to be equally distributed between the vapor and aerosol phases of breath, the concentration of exhaled aerosols would need to be 6.0 *μ*g·m^–3^. Based on commonly used definitions of volatility in the field of atmospheric chemistry [21], we can now classify THC, CBD, and CBN. These C* values put all three cannabinoids in the middle of the range for SVOCs (with C* values from 0.3 to 300 *μ*g·m^–3^). Based on this simple classification scheme, all three cannabinoids are expected to have substantial fractions in both the aerosol and vapor phases. However, given that *p*_corr_ (and, thus C*) is highly dependent on temperature, the partitioning distribution could be impacted by ambient temperature during field testing of breath.

As mentioned above, if the concentration of organic aerosol (*C*_OA_) is known, C* can be used to predict the fraction of an organic compound that resides in the organic aerosol phase (*F*_OA_) with equation (6),

\begin{align*}{F_{{\mathrm{OA}}}} = {\left( {1 + \frac{{C*}}{{{C_{{\mathrm{OA}}}}}}} \right)^{ - 1}}.\end{align*}

Applied to exhaled breath, C* is the value at breath temperature (307.15 K, calculated in the previous paragraph), and *C*_OA_ is the concentration of aerosol in units of *μ*g·m^–3^. Based on previous literature [7, 8], we estimate that a typical concentration of breath aerosol is 1.7 *μ*g·m^–3^. However, concentrations of breath aerosols can vary by an order of magnitude or more [51]. Additionally, aerosols from the environment (such as those from combustion) that are inhaled also contribute to *C*_OA_. The concentration of such ambient aerosols often exceeds those of breath aerosols. For example, the World Health Organization’s air quality guidelines [52] state that 24 hr average exposure to PM2.5 (particulate matter with a diameter of ⩽2.5 *μ*m) should not exceed 15 *μ*g·m^–3^. Particles in this size range have a low probability of deposition in the lungs [9, 53], which means that they contribute to *C*_OA_ and thereby suppress partitioning into the vapor phase (equation (6)).

If *C*_OA_ is assumed to have a typical concentration for breath aerosol alone (1.7 *μ*g·m^–3^), *F*_OA_ is 0.22 for THC, 0.12 for CBD, and 0.38 for CBN. In other words, for all three cannabinoids, the major fraction is predicted to be in the vapor phase. Such a scenario would apply, for example, if a person is breathing filtered air. On the other hand, if the person is breathing untreated air, *C*_OA_ could easily be an order of magnitude higher (17 *μ*g·m^–3^) due to ambient aerosols. In this scenario, the major fraction of all three cannabinoids is predicted to be in the aerosol phase, with *F*_OA_ values of 0.74 for THC, 0.57 for CBD, and 0.86 for CBN. In short, ambient aerosols can directly impact the results of breath sampling for SVOCs.

## 5. Future directions

Regardless of uncertainty in the partitioning predictions due to variability in *C*_OA_, and ignoring the kinetics of partitioning [54], these calculations illustrate an important point: a significant fraction of the mass of THC is predicted to reside in the *vapor phase* of exhaled breath. This suggests that breath collection techniques that only focus on collection of aerosols may be missing a significant fraction of THC and other cannabinoids that are found in breath. It also highlights the perils of using terms like ‘nonvolatile’, which is common in the breath sampling field. As demonstrated by the aerosol-vapor partitioning model, and the volatility classifications from the atmospheric chemistry field, none of the cannabinoids is ‘nonvolatile’ in the sense that it could never be found in the vapor phase. Direct measurements of the partitioning of cannabinoids between the vapor and aerosol phases are warranted. Additionally, further exploration of breath sampling and analysis methods that focus on the vapor phase of exhaled breath is needed.

Our vapor pressure measurements on cannabinoids have led us to question the common assumption that other drug molecules can be treated as ‘nonvolatile’ and therefore must be present only in the breath aerosol phase. For example, vapor pressure measurements may be able to explain why methadone (C_21_H_27_NO, 309.45 g⋅mol^−1^) is widely observed in breath aerosols [55–57], while benzoylecgonine (C_16_H_19_NO_4_, 289.22 g⋅mol^−1^) is rarely detected [55, 58, 59]. In short, future work on the detection of drugs in breath would benefit greatly from low-uncertainty vapor pressure measurements.

## Data Availability

All data that support the findings of this study are included within the article (and any supplementary files). Supplementary material available at https://doi.org/10.1088/1752-7163/ae3794/data1.
